# Educational or Behavioural Interventions to Improve Long‐Term Haemodialysis Vascular Access Self‐Management: A Systematic Review

**DOI:** 10.1111/jorc.70005

**Published:** 2025-01-28

**Authors:** Colette Wembenyui, Nicole Marsh, Emily Larsen, Ann Bonner

**Affiliations:** ^1^ School of Nursing and Midwifery Griffith University Brisbane Queensland Australia; ^2^ Kidney Health Service Royal Brisbane Women's Hospital Herston Queensland Australia; ^3^ Nursing and Midwifery Research Centre Royal Brisbane Women's Hospital Herston Queensland Australia

**Keywords:** chronic kidney disease, haemodialysis, kidney failure, long‐term vascular access, self‐management

## Abstract

**Background:**

Globally, haemodialysis is the most frequent type of kidney replacement therapy and necessitates access to the bloodstream either through a native arteriovenous fistula, arteriovenous graft or central venous catheter. Vascular access complications are a major cause of morbidity and mortality in adults receiving haemodialysis, and effective vascular access self‐management is required.

**Objective:**

To examine the effectiveness of educational or behavioural interventions designed to improve self‐management of long‐term vascular access in adults receiving haemodialysis.

**Design:**

Systematic review.

**Method:**

English language publications from January 2013 to May 2023 retrieved from PubMed, Embase, CINAHL, Cochrane Library, PsycINFO and Joanna Briggs Institute (JBI) databases were undertaken. Two independent reviewers identified studies for full‐text review, data extraction and quality assessment. Data synthesis and quality assessment followed the JBI guidelines for quantitative reviews.

**Results:**

Seven studies involving 540 participants were included: two studies were randomised control trials and five were quasiexperimental. All studies involved patient education, predominantly provided by nurses, and employed a variety of teaching resources, such as education booklets, practical demonstrations and videos. Outcomes measured included vascular access self‐management behaviours, self‐efficacy and vascular access knowledge although there was no consistency between studies. Overall, vascular access self‐management significantly improved following education.

**Conclusion:**

Educational interventions led to improvements in self‐management behaviours in adults with long‐term haemodialysis vascular access. However, there was insufficient evidence for the delivery and duration of intervention education. Further research is needed. An evidence‐based nurse‐led codesign intervention could lead to improvements in vascular access self‐management.

## 1 Introduction

Chronic kidney disease (CKD) is a major healthcare burden, with a global prevalence of over 10% of the world's population (Kovesdy 2022). It is an insidious disease associated with a high incidence of morbidity and mortality (Jha et al. 2023). Kidney failure is the most severe form of CKD, occurring when kidney function (glomerular filtration rate < 15 mL/min per 1.73 m^2^) has deteriorated to the point that it is unable to sustain life (Bello et al. 2022). For people with kidney failure, kidney replacement therapy (dialysis and transplantation) is required for survival.

Haemodialysis (HD) is the most frequently used type of kidney replacement therapy globally (Bello et al. 2022). To provide HD, access to the vascular system is needed; without it, HD treatment cannot be provided, and death would occur. There are three types of vascular access: native arteriovenous fistula (AVF), arteriovenous graft (AVG) and a central venous catheter (CVC). The AVF is preferred for its longevity and lowest association with morbidity and mortality in comparison to the other types of vascular access (Agarwal, Boubes, and Haddad 2021; Canaud et al. 2019). CVCs are used primarily for temporary or short‐term vascular access; however, when an AVF or AVG cannot be established, CVCs are used for long‐term HD (Canaud et al. 2019).

Vascular access management is challenging, requiring a multifaceted approach which includes education, support, collaboration and ongoing communication with patients, their families and the multidisciplinary team (Stavert et al. 2022). To reduce the rate of complications, individuals must perform self‐management actions directed towards HD vascular access maintenance.

Self‐management refers to the active participation of an individual living with a chronic condition in the day‐to‐day management of their disease (Lorig and Holman 2003; Novak et al. 2013). Self‐management is used interchangeably with self‐care in the literature (Lawless et al. 2021), and according to the World Health Organisation (2024), self‐care is “the ability of individuals, families and communities to promote and maintain their own health, prevent disease, and to cope with illness—with or without the support of a health or care worker”. For the purpose of this review, we refer to both concepts as self‐management because both indicate the active involvement of a person in their chronic disease management (Lorig and Holman 2003; Orem 1991).

Effective self‐management is required for people with CKD and there are specific and additional self‐management activities needed to maintain HD vascular access. These activities include monitoring its patency (e.g., the presence of thrill over the fistula), avoiding restrictive garments, lying down over the fistula arm, blood pressure checking and using the fistula limb to carry excess weight, venipuncture or medication infusion on the fistula limb (Liu et al. 2016). Teaching people how and why to self‐manage their vascular access is key to monitoring and preventing complications. Nurses have a key role in supporting patients to self‐manage by providing education aimed at fostering the acquisition of knowledge and skills required to care for their vascular access (Dineen‐Griffin et al. 2019). This support may involve asking about how often they check for the presence of thrill (vibration of the fistula); for CVC monitoring insertion site for signs of infection (e.g., redness, swelling or pain) and providing guidance on the frequency of checks; asking patients to ensure that needle sites are rotated during cannulation to prevent stenosis and asking and acknowledging their opinions about their vascular access. These interactions provide opportunities for patients to be empowered to self‐manage their vascular access.

There have been three reviews specifically about HD self‐management. The first was a discursive review and included six studies published between 2000 and 2010, although this review did not report on interventions to promote vascular access self‐management (Sousa et al. 2014). The second review (Costa Pessoa et al. 2020) included 15 studies, published between 2013 and 2018, although none involved self‐management education interventions. A recent systematic review of knowledge of AVF self‐management (Natti Krishna et al. 2023) has significant limitations. For example, all seven studies in this review were from one geographical region (south Asian countries) and of cross‐sectional design to determine the level of knowledge about AVF self‐management. No studies involved interventions to improve knowledge. Meta‐analysis was similarly flawed because knowledge was not measured homogeneously. To mitigate heterogeneity, the authors reduced the number of studies included in the meta‐analysis (Natti Krishna et al. 2023). Although these systematic reviews exist, none involved interventions targeting vascular access self‐management. Thus, this systematic review is needed to assess evidence of educational or behavioural interventions designed to improve HD vascular access self‐management.

The aim of this systematic review was to evaluate the effectiveness of educational or behavioural interventions to improve long‐term vascular access in adults receiving HD. The review sought to answer the following research questions:

- What educational or behavioural interventions were used to teach HD vascular access self‐management?
- How effective are educational or behavioural interventions compared with routine care at improving long‐term HD vascular access self‐management?
- What instruments have been used to measure long‐term HD vascular access self‐management in adults receiving haemodialysis?
- What instruments were used to determine the effectiveness of the educational or behavioural interventions?

## 2 Methods

### 2.1 Design

This systematic review followed the Joanna Briggs Institute (JBI) guidelines (Tufanaru et al. 2020) and the Synthesis without Meta‐analysis (SWiM) reporting guidelines (Campbell et al. 2020). The review protocol was prospectively registered in PROSPERO (CRD42023414193).

### 2.2 Search Strategy

An initial search was conducted in PubMed and Embase to identify keywords related to HD, vascular access, self‐management and patient education, followed by analysing titles, abstracts and index terms. Since both self‐care and self‐management are used in the literature, both were included as search terms. Using these keywords, six databases (PubMed, Embase, CINAHL, APA PschINFO, The Cochrane Library and JBI) were searched in May 2023 for studies published in English between January 2013 and May 2023. Medical subject headings (MeSH) (e.g., “Renal Dialysis” OR “Haemodialysis” OR “Kidney Replacement Therapy” AND “Vascular Access Devices” OR “Arteriovenous Fistula” OR “Central Venous Catheter” AND “Education” OR “ Self‐Care” OR “Self‐Management”) and keywords (e.g., kidney failure, end‐stage kidney disease, dialysis, knowledge, vascular access, self‐care and self‐management) were combined using Boolean, truncation and wildcard operators for the search (see Supporting Information File 1). Lastly, the reference lists of all included articles were hand searched for any additional studies.

### 2.3 Inclusion and Exclusion Criteria

This review included randomised controlled trials (RCTs), pseudo‐RCTs and quasiexperimental studies of adults aged 18 years or over, with kidney failure who had vascular access required for chronic long‐term HD. Exclusion criteria were predialysis, emergency‐start dialysis participants or transplant recipients. We restricted the search to published articles written in English.

This review considered studies that evaluated educational or behavioural interventions designed to improve long‐term HD vascular access self‐management. Studies that investigated different types of educational or behavioural methods used alone or in combination, either in routine practice or research interventions were included in this review. There were no restrictions on study duration, or whether a follow‐up assessment was conducted. The practice or intervention could be delivered by any health professional.

### 2.4 Selection of Studies

**Figure 1:**
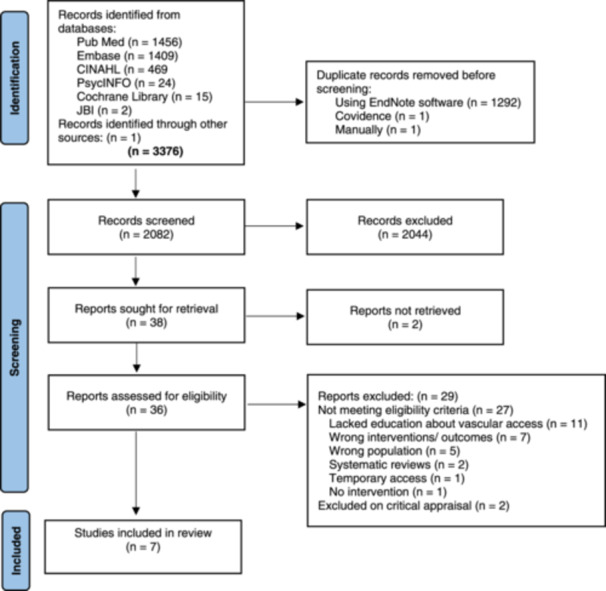
Preferred reporting items for systematic review and meta‐analysis flow diagram of included studies. *Source:* Page et al. (2021). JBI, Joanna Briggs Institute.

Following database searches, all identified studies were exported into Covidence (Veritas Health Innovation, Melbourne, Australia). After the removal of duplicates, two reviewers independently (C.W., with either E.L. or N.M.) screened the title and abstracts for study inclusion. Full texts of eligible articles were retrieved and again independently reviewed by two study authors (C.W. and N.M.) for inclusion. A third reviewer's (A.B.) judgement was sought for disagreements to reach a consensus. The study selection process is shown in Figure 1 using the Preferred Reporting Items for Systematic Review and Meta‐Analysis flow diagram (Page et al. 2021).

### 2.5 Data Extraction and Quality Appraisal

Two study authors (C.W. and N.M.) independently extracted data using a customised data extraction form which included study authors, year of publication, country, study setting, sample size, study design, intervention, comparator, outcome measured, key findings, study strength and limitation (Table 1). Any disagreements were resolved by a third author (A.B.). Information on intervention delivery mode, programme duration and follow‐up periods were extracted (Table 2). Data about the instruments used to measure the study outcomes were also extracted and tabulated (Table 3).

**Table 1:** Characteristics of included studies.

Author (year), country	Study design	Sample characteristic Age (Mean ± SD)	Theoretical framework	Intervention/control	Outcomes/key findings	Strength/limitations
Borzou et al. (2020), Iran	Quasiexperimental study	Teach‐back group (*n* = 34) Age: 52 ± 12.9 Male (%): 55.9 Group discussion group (*n* = 33) Age: 52.7 ± 12.6 Male (%): 69.7	No	Teach‐back group Nutrition, activity‐rest and fistula care. Group discussion group Same topics as the teach‐back group.	*Outcomes:* Knowledge, attitude and performance. *Key findings:* Knowledge, attitude and performance increased in both groups postintervention (*p* < 0.001) although, mean attitude was higher in the teach‐back group (*p* < 0.01).	*Strength* Used the teach‐back method of teaching. Confounding variables were controlled. The sample was powered. *Limitation* No control group. Change in self‐management not measured. Unclear intervention fidelity.
Fadlalmola and Elkareem (2020), Sudan	Quasiexperimental study	*n* = 100 Age (Mean): 50 Male (%): 59	No	Education programme Concept of HD, vascular access care, complications, dietary and fluids restrictions, medications, activities for disease and HD adaptability.	*Outcomes*: Knowledge and HRQoL. *Key findings*: Significant increase in overall knowledge, including fistula and vascular access self‐management (*p* < 0.05). Significant improvement in all domains of HRQoL (*p* < 0.05).	*Strength* Changes in knowledge measured. Sample size calculation shown. *Limitations* Incomplete demographic data. No control group. Unclear intervention fidelity. Changes in self‐management not measured.
Li and Yin (2021), China	Quasiexperimental study	Intervention group (*n* = 50) Male (%): 70 Control group (*n* = 50) Male (%): 60	No	Intervention Self‐management education and self‐protection behaviour education. Control Routine nursing care.	*Outcomes*: Self‐management ability, internal fistula quality. *Key findings*: Significant improvement in self‐management ability (*p* < 0.05) and internal fistula quality (*p* < 0.001) in the intervention group compared to the control group.	*Strength* Changes in self‐management measured. *Limitations* Sample not powered. Unclear methods. Limited demographic data. Unclear intervention fidelity.
Liu et al. (2016), China	Randomised controlled trial	Intervention group (*n* = 43) Age: 41.7 ± 15.8 Male (%): 53.5 Control group (*n* = 43) Age: 44.3 ± 14.6 Male (%): 60.5	Kelman's change of attitude theory	Intervention group Knowledge–attitude–behaviour health education model. Control group Routine health education on diet, medication, exercise, correct fistula care disease condition monitoring.	*Outcomes:* Disease knowledge and self‐management behaviour. *Key findings*: Significantly higher knowledge scores and self‐management behaviours in the intervention group compared to the control group (*p* < 0.001).	*Strength* Changes in self‐management and knowledge measured Intervention based on a theoretical framework. *Limitations* No mention of blinding and concealing. Intention to treat not mentioned.
Choudhury et al. (2019), Iran	Randomised controlled trial	Intervention group (*n* = 35) Age: 41.8 0 ± 9.68 Male (%): 57.1 Control group (*n* = 35) Age: 43 ± 11.65 Male (%): 74.3	Self‐efficacy theory	Intervention group General information about CKD, adherence to diet and fluid restrictions, skin and fistula care, physical activity and rest and educational packs were given. Control group Routine dialysis care.	*Outcomes:* Self‐efficacy. Awareness and self‐management dimensions. *Key finding:* Significant increase in awareness, self‐efficacy, and self‐management in the intervention group compared to the control group (*p* < 0.001).	*Strength* Changes in self‐management measured. Intervention based on a theoretical framework. *Limitations* Sample not powered. No mention of blinding and concealing. Intention to treat not mentioned.
Sousa et al. (2021), Portugal	Quasiexperimental study	Intervention group (*n* = 48) Age: 58.7 ± 14.3 Male (%): 64.6 Control group (*n* = 41) Age: 65.8 ± 13.9 Male (%): 65.9	No	Intervention group Structured intervention for AVF self‐management. Control group Usual care, which involved education on AVF care and occasional training with no plan in place.	*Outcomes*: Frequency of self‐management behaviours with AVF. *Key findings:* Better overall self‐management behaviours with AVF in the intervention group compared to the control group (*p* < 0.001).	*Strength* Changes in self‐management measured. *Limitations* Sample not powered. Risk of sampling bias, control and intervention group not similar at baseline.
Trask et al. (2016), Canada	Quasiexperimental study	*n* = 28	No	Intervention Written orientation educational booklet. Individually tailored face‐to‐face orientation. The Bridge Curriculum. Self‐management ability documentation tool.	*Outcomes*: Self‐management related to weight gain, vascular access and fluid intake and diet. *Key findings*: Significant improvement in self‐management of vascular access (*p* < 0.002) and fluid management (*p* < 0.1) Nonsignificant changes in interdialytic weight gain and diet.	*Strength* Changes in self‐management measured. *Limitations* Small group sizes. Limited demographic data. No control group.

Abbreviations: AVF, arteriovenous fistula; BP, blood pressure; CKD, chronic kidney disease; GAP, generally acceptable practice; HD, haemodialysis; HRQoL, health‐related quality of life.

**Table 2:** Teaching plan for education interventions.

Author (year)	Intervention duration	Number of sessions	Length of each session	Educator	Mode of delivery	Timing of intervention	Post‐intervention follow‐up
Borzou et al. (2020)	2 weeks	3	30–45 min	Researcher	Individual face‐to‐face teach‐back, small group discussion	After HD treatment	1 month
Fadlalmola and Elkareem (2020)	3 months	Not reported	Not reported	Nurse	Education programme	Not reported	6 months
Li and Yin (2021)	> 3 months	Not reported	Not reported	Nurse	Group education sessions, including self‐protection behaviour	Not reported	3 months
Liu et al. (2016)	Not reported	Not reported	Not reported	Nurse	Face‐to‐face group discussions with individualised information, if necessary, telephone	Not reported	6 months
Choudhury et al. (2019)	2 weeks	4	1 h	Researcher	Face‐to‐face group training sessions, education pamphlets	Nondialysis days	3 months
Sousa et al. (2021)	2 days (theory)	6	30 min	Researcher	Face‐to‐face group presentations	Before HD session	3 months
	2 weeks (practical)	2	15 min	Nurse and Researcher	Interactive small group training sessions.	At the start of HD sessions	
Trask et al. (2016)	Not reported	Not reported	Not reported	Nurse	Face‐to‐face education, orientation document	During HD treatment	1 year

Abbreviation: HD, haemodialysis.

**Table 3:** Instruments used to measure intervention outcomes (*n* = 11).

Author (year)	Instrument	Outcome measured	Items/score range	Scoring/content	Validity and reliability
Borzou et al. (2020)	Researcher‐made questionnaire	Knowledge, attitude and performance of people receiving HD	Knowledge (19 items), scoring 0 or 1; attitude (26 items) and performance (25 items) both scored on a five‐point Likert scale	Not reported	CVR = 0.84 CVI = 0.81 ICC = 0.911
Fadlalmola and Elkareem (2020)	Researcher‐made questionnaire	Knowledge about HD and vascular access	Not reported	Not reported	Cronbach's alpha 0.84
Li and Yin (2021)	Self‐management scale	Self‐management ability	20 items, scoring on a four‐point Likert scale	Higher scores indicate better self‐management	Not reported
	Internal fistula quality assessment scale	Internal fistula quality	Scoring: excellent, good, or poor	Not reported	Not reported
Liu et al. (2016)	Chronic disease self‐management scale	Self‐management behaviour	20 items, scoring on a four‐point Likert scale	Not reported	Cronbach's alpha 0.89
	Disease knowledge questionnaire	Disease knowledge	20 items, score range: 0–3	Higher scores indicate better self‐management behaviour	Not reported
Choudhury et al. (2019)	SUPPH	Self‐efficacy	29 items with four dimensions. Score range 1–5	Not reported	Cronbach's alpha 0.91
	Researcher‐made questionnaire	Self‐awareness	10 items with “True” or “False” answers	Not reported	CVR > 0.89 CVI = 0.94
		Self‐management	40 items, scoring on a five‐point Likert scale	Not reported	
Sousa et al. (2021)	ASBHD‐AVF	Self‐management behaviours with AVF	16 items with two subscales. Scoring, on a five‐point Likert scale	Higher scores indicate a higher frequency of AVF self‐management	Cronbach's alpha 0.80
Trask et al. (2016)	SCAD	Self‐management abilities and behaviours	66 items with five domains. Scoring, on a five‐point Likert scale	Higher scores indicate greater self‐management abilities and behaviours	Cronbach's alpha 0.95

Abbreviations: ASBHD‐AVF, scale of assessment of self‐care behaviours with arteriovenous fistula in haemodialysis; CVI, content validity index; CVR, content validity ratio; HD, haemodialysis; ICC, intraclass correlation coefficient; SCAD, self‐care for adults on dialysis tool; SUPPH, strategies used by people to promote health.

The JBI standardised critical appraisal tools for RCT, and quasiexperimental studies guided quality assessment of eligible studies (Supporting Information File 2). Two reviewers (C.W. and N.M.) independently assessed the quality of each study to reduce the risk of bias. A third reviewer (A.B.) was consulted where there was disagreement to reach a consensus.

### 2.6 Data Synthesis

Data synthesis followed the JBI guidelines for quantitative reviews (Tufanaru et al. 2020). A meta‐analysis was not conducted due to the heterogeneity of study participants, outcomes and measuring instruments, therefore SWiM was conducted instead.

## 3 Results

### 3.1 Characteristics of Included Studies

Initially, 3376 studies were identified; following removal of duplicates, 2082 studies were screened. Seven studies were retained for this review after a full‐text review and quality assessment (Figure 1). Most of the study designs were quasiexperimental (Fadlalmola and Elkareem 2020; Li and Yin 2021; Sousa et al. 2021; Trask et al. 2016), while the remaining two studies were RCTs (Liu et al. 2016; Choudhury et al. 2019). Included studies were from China (*n* = 2), Iran (*n* = 2), Canada (*n* = 1), Portugal (*n* = 1) and Sudan (*n* = 1), involving a total of 540 participants. Six studies included participants who had been on HD for at least 3 months (Borzou et al. 2020; Fadlalmola and Elkareem 2020; Li and Yin 2021; Liu et al. 2016; Sousa et al. 2021; Trask et al. 2016); however, one study did not report study participants duration of HD (Choudhury et al. 2019). The sample sizes ranged from 28 (Trask et al. 2016) to 100 participants (Fadlalmola and Elkareem 2020; Li and Yin 2021). Over 50% (*n* = 289) of the study participants were males (Borzou et al. 2020; Fadlalmola and Elkareem 2020; Li and Yin 2021; Liu et al. 2016; Choudhury et al. 2019; Sousa et al. 2021). Participants' mean age ranged from 41.7 (Liu et al. 2016) to 65.8 years (Sousa et al. 2021), although two studies (Li and Yin 2021; Trask et al. 2016) did not report participants' mean age. The characteristics of included studies are presented in Table 1.

Study quality scores ranged from 55% to 100%, with most studies rated as having moderate methodical quality (Fadlalmola and Elkareem 2020; Li and Yin 2021; Liu et al. 2016; Choudhury et al. 2019). Detailed scoring of methodology quality of included studies is presented in Supporting Information File 2.

### 3.2 Educational and Behavioural Interventions

All seven studies employed education interventions (Table 2), although only two studies were informed by theoretical frameworks; Kelman's change of attitude theory (Liu et al. 2016) and self‐efficacy theory (Choudhury et al. 2019). Most of the interventions were delivered face‐to‐face either through oral presentations or small group discussions (Borzou et al. 2020; Liu et al. 2016; Choudhury et al. 2019; Sousa et al. 2021; Trask et al. 2016). The studies used a variety of learning resources, such as education booklets, orientation documents, practical demonstrations, telephone calls and video films (Liu et al. 2016; Choudhury et al. 2019; Sousa et al. 2021; Trask et al. 2016). Participants who had acquired adequate self‐management behaviours were allocated to different groups to serve as role models and encouraged other participants by sharing their personal experiences (Choudhury et al. 2019). Borzou et al. (2020) was the only study that used the teach‐back method to deliver the education intervention. Teach‐back is a technique of checking understanding in which the healthcare provider asks the patient or their family to repeat the information that has been provided to them so that they can correct any misunderstanding (Yen and Leasure 2019).

The education interventions were given at varying times, such as before or at the start of HD (Sousa et al. 2021), during HD (Trask et al. 2016), after HD (Borzou et al. 2020) or on nondialysis days (Choudhury et al. 2019). Three studies did not report on the timing of the educational intervention delivery (Fadlalmola and Elkareem 2020; Li and Yin 2021; Liu et al. 2016). Most of the studies delivered self‐management education about vascular access as a topic in a broader educational intervention. Only two studies focused specifically on AVF self‐management behaviours (Li and Yin 2021; Sousa et al. 2021).

Intervention durations varied from 2 weeks (Borzou et al. 2020; Choudhury et al. 2019; Sousa et al. 2021) to 3 months (Fadlalmola and Elkareem 2020). Three studies did not report intervention duration (Li and Yin 2021; Liu et al. 2016; Trask et al. 2016). Of the studies that reported the number and length of sessions, the number of education sessions ranged from three (Borzou et al. 2020) to eight sessions (Sousa et al. 2021), and the length of each session varied from 15 min (Sousa et al. 2021) to 1 h (Choudhury et al. 2019). Sousa et al. (2021) delivered the education intervention in two components: a theoretical and a practical component. The theoretical component involved six sessions about the care of an AVF, delivered to small groups of participants over 2 days. The practical component, which was conducted 1 week after the theoretical component, involved interactive sessions teaching participants about inspection and palpation skills of an AVF. Follow‐up periods ranged from 1 month (Borzou et al. 2020) up to 1 year (Choudhury et al. 2019).

Education interventions were delivered by nurses (Fadlalmola and Elkareem 2020; Li and Yin 2021; Liu et al. 2016; Sousa et al. 2021; Trask et al. 2016) or the researcher (Borzou et al. 2020; Choudhury et al. 2019; Sousa et al. 2021); it is unclear whether the researchers were also nurses. Vascular access topics covered were protection from trauma, checking for thrill, cleansing site before HD, monitoring for infection (Li and Yin 2021; Liu et al. 2016; Sousa et al. 2021; Trask et al. 2016), holding fistula sites, doing limb exercises (Trask et al. 2016), not weight bearing on the fistula limb and not using it for venesection, blood pressure measurements, injections or infusions (Liu et al. 2016). Three studies did not report on the type of vascular access education provided (Borzou et al. 2020; Fadlalmola and Elkareem 2020; Choudhury et al. 2019).

Two studies evaluated the effectiveness of an AVF self‐management intervention (Li and Yin 2021; Sousa et al. 2021) and reported significant improvements in the self‐management behaviours of participants who received the intervention. Education intervention improved the development of skills to monitor and identify AVF complications (Sousa et al. 2021). Similarly, the other studies that measured self‐management behaviours reported significant improvements in self‐management following an education intervention (Liu et al. 2016; Choudhury et al. 2019; Trask et al. 2016). Choudhury et al. (2019) also measured self‐efficacy and found a significant increase in self‐efficacy scores following the education intervention. Self‐efficacy refers to an individual's belief in their capacity to accomplish a specific behaviour; it reflects confidence in their ability to perform an action (Bandura 1986).

Three studies measured knowledge about fistula/CVC care and found significant improvements in knowledge postintervention (Borzou et al. 2020; Fadlalmola and Elkareem 2020; Liu et al. 2016); however, Borzou et al. (2020) found that between group analysis was not significant, indicating that group discussion and teach‐back self‐management education had a similar effect on knowledge. None of the studies included in this review provided predialysis AVF self‐management education.

### 3.3 Instruments Used to Measure Study Outcomes

Ten instruments were used to measure intervention outcomes (Table 3). Of these, only one instrument was specific for vascular access self‐management (Sousa et al. 2021). Three instruments were developed by the researcher and these measured: knowledge, attitude and performance (Borzou et al. 2020), knowledge about HD and vascular access (Fadlalmola and Elkareem 2020) and self‐awareness and self‐management (Choudhury et al. 2019). Other instruments used were diverse in nature and measured self‐management ability and AVF quality (Li and Yin 2021); self‐management behaviour and disease knowledge (Liu et al. 2016); self‐efficacy (Choudhury et al. 2019); AVF self‐management behaviours (Sousa et al. 2021); and self‐management abilities and behaviours (Trask et al. 2016).

## 4 Discussion

Despite the critical role of vascular access for adults receiving HD, this review identified only seven studies, which assessed interventions to support essential HD self‐management. Of these, only two were specific for HD vascular access self‐management and none of the studies involved behavioural interventions. Overall, the methodological quality of the included studies was moderate. There was, however, substantial heterogeneity between studies and as such the results of this systematic review should be interpreted with caution. While five studies included in this review showed improvements in HD vascular access self‐management behaviours (Li and Yin 2021; Liu et al. 2016; Choudhury et al. 2019; Sousa et al. 2021; Trask et al. 2016), none of the study samples were powered to determine statistical significance.

Improvements in self‐management behaviours were largely generalised, making it difficult to ascertain which vascular access self‐management behaviours needed more attention. One study did acknowledge that while monitoring of vascular access sites improved following education, this behaviour was not sustained over time (12‐month period) (Trask et al. 2016). Education to improve vascular access self‐management was mostly given as part of a broader education intervention, and only two studies in this review focused on AVF self‐management (Li and Yin 2021; Sousa et al. 2021). According to Dineen‐Griffin et al. (2019), education to improve self‐management provides adults with skills to monitor their own health and to problem‐solve. One study in this review revealed that individuals who received educational intervention were better at identifying potential problems with their AVF and seeking medical assistance (Sousa et al. 2021). The results of this review suggest education interventions may improve HD vascular access self‐management. However, there is limited evidence about education interventions to support these behaviours. Studies examining the effectiveness of self‐management intervention programmes in adults with CKD found that increasing self‐efficacy led to improvements in self‐management behaviours (Chuang et al. 2021; Curtin et al. 2008; Havas, Douglas, and Bonner 2018; Milazi, Douglas, and Bonner 2018; Nguyen, Douglas, and Bonner 2019). One study in this review found that education to increase self‐efficacy might be effective in improving AVF self‐management (Choudhury et al. 2019), and further research is needed.

We also found that educational interventions led to a significant increase in knowledge about vascular access maintenance and care. A meta‐analysis by Milazi, Bonner, and Douglas (2017) found that educational or behavioural interventions significantly improved knowledge of phosphate control in adults receiving HD. Similar results have also been found in other studies assessing the impact of education intervention on knowledge in the HD population (Alikari et al. 2019; Dsouza et al. 2023). Knowledge is an important precursor for behaviour change (Arlinghaus and Johnston 2018). Despite this, interventions to improve knowledge about vascular access self‐management are scarce. When patients have a sufficient level of understanding about vascular access care and maintenance, they are more likely to care for their vascular access.

The duration, mode of delivery, frequency and timing of education are core elements of effective education (Phillips et al. 2014). Despite this, our review demonstrated insufficient evidence for the delivery and duration of intervention education, and teaching resources used. Dineen‐Griffin et al. (2019) suggest that patient‐centred multicomponent educational interventions delivered face‐to‐face either individually or in groups could lead to increased self‐management behaviours. In our review, Li and Yin (2021) delivered education intervention through group presentations, while Sousa et al. (2021) employed a structured intervention that consisted of face‐to‐face group presentations and interactive group training sessions. Similarly, Odgers‐Jewell et al. (2017) found education provided in small groups improved learning through shared experiences, and enhanced motivation and self‐efficacy. Thus, these core elements of effective education should be considered a high priority for future research.

This review did not find studies using behavioural interventions such as motivational interviewing or empowerment for self‐management. Motivational interviewing is a patient‐centred counselling approach to behaviour change that acknowledges a person's reasons for change and is goal‐orientated (Ok and Kutlu 2021; Papus et al. 2022). One of the central principles of motivational interviewing is to support self‐efficacy. It has been used to increase treatment adherence and quality of life in adults receiving HD (Ok and Kutlu 2021). However, the one study (Choudhury et al. 2019) in this review that assessed self‐efficacy did not use motivational interviewing. Providing education alone is not enough for behaviour change. To elicit behaviour change, education should be given to increase awareness of why behaviour change is necessary and training on how to bring about the change (Arlinghaus and Johnston 2018). Motivational interviewing could be used as an approach to improve HD vascular access self‐management, as it increases both awareness and self‐management skills.

This review did not identify any study that reported providing education about vascular access prior to its creation. In a scoping review, Van Eck Van Der Sluijs et al. (2021) highlight the importance of providing comprehensive predialysis education that includes vascular access and ongoing self‐management education to help improve outcomes and quality of life in adults receiving HD. Predialysis education about vascular access is aimed at preserving the vascular network, such as avoiding venous punctures on the limb mapped for AVF creation (Sousa et al. 2014). Exercise of fistula limb before access creation increases blood vessel size, which may facilitate the creation of a distal AVF (González et al. 2023; Kumar A/L S Katheraveloo et al. 2020) and consequently favour better maturation (Nantakool et al. 2022). Priority should be given to education about HD vascular access before its creation and vascular access self‐management.

### 4.1 Strengths and Limitations

This is the first review on the effectiveness of educational or behavioural interventions on vascular access self‐management behaviours in adults receiving HD. This review also adhered to the robust process of conducting a systematic review by following the JBI and SWiM reporting guidelines. This review, however, also had several limitations. First, most of the studies included in this review provided education strategies about vascular access self‐management as part of a larger intervention. Also, improvements in vascular access self‐management were reported as generalised statements making it difficult to determine which areas of education needed to be reinforced. The included studies were heterogeneous, with differences in design, delivery, intensity and instruments used to measure outcomes, which prevented a meta‐analysis of study outcomes from being conducted. Lastly, only articles published in the English language within a 10‐year period were considered for this review; some studies might have been missed.

## 5 Conclusion

This review synthesised current evidence on the effectiveness of educational or behavioural interventions on long‐term HD vascular access self‐management. Regardless of whether the term self‐care or self‐management was used, the studies included in this review employed similar educational strategies to support behaviours with long‐term HD vascular access. Education interventions improved vascular access self‐management in adults receiving HD. Given the significance of a functioning vascular access for the delivery and effectiveness of HD treatments, it is necessary that education about vascular access should be given before its creation and should remain an important component of ongoing HD care. Current evidence for educational or behavioural interventions to support HD vascular access self‐management is limited. Further interventional research is needed.

### 5.1 Implication for Clinical Practice

Vascular access is a patient's lifeline and affects both the ability to provide HD as well as the quality of HD treatment itself. This review only found limited evidence on which to guide practice; high‐quality evidence‐based studies on educational or behavioural interventions to improve vascular access self‐management and inform nursing practice are urgently needed. Despite there being limited evidence to support when to give education, it is recommended that education should begin before the creation of vascular access and then be reinforced afterwards to promote maturation and preservation of the access. The principles of codesign could be used to develop person‐centred strategies to improve vascular access self‐management. Nurses have extensive contact with people receiving HD and are in an ideal situation to provide information and education needed for self‐management, especially about vascular access. It is important to be able to measure the level of self‐management to provide targeted education to improve vascular access self‐management. Validated and reliable instruments that measure HD vascular access self‐management are warranted. We recommend that appropriate training be provided to dialysis nursing staff about teaching vascular access self‐management.

## Author Contributions

**Colette Wembenyui:** conceptualisation, methodology, formal analysis, writing–original draughting of the manuscript writing–review and editing, and visualisation. **Nicole Marsh:** conceptualisation, methodology, formal analysis, writing–review and editing, and supervision. **Emily Larsen:** methodology, formal analysis and writing–review and editing. **Ann Bonner:** conceptualisation, methodology, formal analysis, writing–review and editing, supervision, critical review of intellectual content and final approval of the manuscript.

## Conflicts of Interest

The authors declare no conflicts of interest.

## Supporting information

Supporting information.

Supporting information.
