# Supplementary material for: Educational or Behavioural Interventions to Improve Long‐Term Haemodialysis Vascular Access Self‐Management: A Systematic Review
Source: J Ren Care. 2025 Jan 28;51(1):e70005. doi: 10.1111/jorc.70005 (PMC11774008; doi:10.1111/jorc.70005)
Supplement: Supplementary file 1 — Supporting information. [file JORC-51-0-s002.docx]

**Supplementary File 1: Systematic Review Searches**

**Date searched - 30 May 2023**

**Database searches**

**PubMed** 1456 results

Includes MeSH

**(**"Renal Dialysis"[Mesh] OR "Renal Replacement Therapy"[Mesh] OR "Hemofiltration"[Mesh] OR "Hemodialysis Units, Hospital"[Mesh] OR "Kidney Failure, Chronic"[Mesh] OR "Renal Insufficiency"[Mesh] OR "Renal Insufficiency, Chronic"[Mesh] OR "Kidney"[Mesh] OR “dialysis”[tiab] OR “Renal Dialyses”[tiab] OR "Renal Dialysis"[tiab] OR “Hemodialysis”[tiab] OR “Hemodialyses”[tiab] OR “Haemodialysis”[tiab] OR “Haemodialyses”[tiab] OR “hemofiltration”[tiab] OR “haemofiltration”[tiab] OR “Hemodiafiltration”[tiab] OR “Haemodiafiltration”[tiab] OR “kidney replacement therapy”[tiab] OR “kidney replacement therapies”[tiab] OR “renal replacement therapy”[tiab] OR “renal replacement therapies”[tiab] OR “kidney failure”[tiab] OR “renal failure”[tiab] OR “Chronic Kidney Diseases”[tiab] OR “Chronic Kidney Disease”[tiab] OR “Chronic Renal Diseases”[tiab] OR “Chronic Renal Disease”[tiab] OR “End Stage Kidney Disease”[tiab] OR “End Stage Renal Disease”[tiab] OR “renal insufficiency”[tiab] OR “uraemia”[tiab] OR “uremia”[tiab] OR “azetoma”[tiab] OR “ESKD”[tiab] OR “ESRF”[tiab] OR “ESKF”[tiab] OR “CKD”[tiab] OR “CRF”[tiab] OR “CKF”[tiab] OR “CRD”[tiab] OR “KRT”[tiab] OR “RRT”[tiab]**) AND (**"Vascular Access Devices"[Mesh] OR "Arteriovenous Fistula"[Mesh] OR "Catheterization, Central Venous"[Mesh] OR “vascular access”[tiab] OR “graft”[tiab] OR “grafts”[tiab] OR “fistula”[tiab] OR “fistulas”[tiab] OR “tunnelled catheter”[tiab] OR “tunneled catheter”[tiab] OR “nontunnelled catheter”[tiab] OR “nontunneled catheter”[tiab] OR “non tunnelled catheter”[tiab] OR “non tunneled catheter”[tiab] OR “central venous catheter”[tiab] OR “central venous catheters”[tiab] OR “vascular catheter”[tiab] OR “vascular catheters”[tiab] OR “venous catheter”[tiab] OR “venous catheters”[tiab] OR “central venous catheterization”[tiab] OR “central venous catheterizations”[tiab] OR “central venous catheterisation”[tiab] OR “central venous catheterisations”[tiab]**)** **AND (**"Education"[Mesh] OR "Health Education"[Mesh] OR "Patient Education as Topic"[Mesh] OR "Knowledge"[Mesh] OR "Health Literacy"[Mesh] OR "Self Care"[Mesh] OR "Self-Management"[Mesh] OR "Self Efficacy"[Mesh] OR "Counseling"[Mesh] OR “educational”[tiab] OR “education”[tiab] OR “knowledge”[tiab] OR “train*”[tiab] OR “teach*”[tiab] OR “treatment information”[tiab] OR “health literacy”[tiab] OR “behavioural”[tiab] OR “behavioral”[tiab] OR “behaviour”[tiab] OR “behavior”[tiab] OR “self management”[tiab] OR “self care”[tiab] OR “self efficacy”[tiab] OR “cognitive”[tiab] OR “counselling”[tiab] OR “counseling”[tiab]**)** **AND** (eng[la] OR und[la]) **AND** 2013:2023[dp] **NOT** (animals [mh] NOT humans [mh])

**Embase (Elsevier)** 1409 results

Includes Emtree

**(**'hemodialysis'/exp OR 'renal replacement therapy'/exp OR 'hemofiltration'/exp OR 'chronic kidney failure'/exp OR 'kidney failure'/exp OR 'kidney'/exp OR “dialysis”:ti,ab OR “Renal Dialyses”:ti,ab OR "Renal Dialysis":ti,ab OR “Hemodialysis”:ti,ab OR “Hemodialyses”:ti,ab OR “Haemodialysis”:ti,ab OR “Haemodialyses”:ti,ab OR “hemofiltration”:ti,ab OR “haemofiltration”:ti,ab OR “Hemodiafiltration”:ti,ab OR “Haemodiafiltration”:ti,ab OR “kidney replacement therapy”:ti,ab OR “kidney replacement therapies”:ti,ab OR “renal replacement therapy”:ti,ab OR “renal replacement therapies”:ti,ab OR “kidney failure”:ti,ab OR “renal failure”:ti,ab OR “Chronic Kidney Diseases”:ti,ab OR “Chronic Kidney Disease”:ti,ab OR “Chronic Renal Diseases”:ti,ab OR “Chronic Renal Disease”:ti,ab OR “End Stage Kidney Disease”:ti,ab OR “End Stage Renal Disease”:ti,ab OR “renal insufficiency”:ti,ab OR “uraemia”:ti,ab OR “uremia”:ti,ab OR “azetoma”:ti,ab OR “ESKD”:ti,ab OR “ESRF”:ti,ab OR “ESKF”:ti,ab OR “CKD”:ti,ab OR “CRF”:ti,ab OR “CKF”:ti,ab OR “CRD”:ti,ab OR “KRT”:ti,ab OR “RRT”:ti,ab**) AND (**'vascular access device'/exp OR 'arteriovenous fistula'/exp OR 'central venous catheterization'/exp OR “vascular access”:ti,ab OR “graft”:ti,ab OR “grafts”:ti,ab OR “fistula”:ti,ab OR “fistulas”:ti,ab OR “tunnelled catheter”:ti,ab OR “tunneled catheter”:ti,ab OR “nontunnelled catheter”:ti,ab OR “nontunneled catheter”:ti,ab OR “non tunnelled catheter”:ti,ab OR “non tunneled catheter”:ti,ab OR “central venous catheter”:ti,ab OR “central venous catheters”:ti,ab OR “vascular catheter”:ti,ab OR “vascular catheters”:ti,ab OR “venous catheter”:ti,ab OR “venous catheters”:ti,ab OR “central venous catheterization”:ti,ab OR “central venous catheterizations”:ti,ab OR “central venous catheterisation”:ti,ab OR “central venous catheterisations”:ti,ab**)** **AND (**'education'/exp OR 'health education'/exp OR 'patient education'/exp OR 'knowledge'/exp OR 'health literacy'/exp OR 'self care'/exp OR 'counseling'/exp OR “educational”:ti,ab OR “education”:ti,ab OR “knowledge”:ti,ab OR “train*”:ti,ab OR “teach*”:ti,ab OR “treatment information”:ti,ab OR “health literacy”:ti,ab OR “behavioural”:ti,ab OR “behavioral”:ti,ab OR “behaviour”:ti,ab OR “behavior”:ti,ab OR “self management”:ti,ab OR “self care”:ti,ab OR “self efficacy”:ti,ab OR “cognitive”:ti,ab OR “counselling”:ti,ab OR “counseling”:ti,ab**)** **AND** [english]/lim **AND** [2013-2023]/py **NOT** (‘animal experiment’/de NOT (‘human experiment’/de OR ‘human’/de)) **AND** ([article]/lim OR [article in press]/lim OR [review]/lim)

**CINAHL Complete (EBSCOhost)** 469 results

Includes CINAHL Subject Headings

**(**MH "Renal Replacement Therapy+" OR MH "Hemofiltration+" OR MH "Kidney Failure, Chronic+" OR MH "Renal Insufficiency+" OR MH "Renal Insufficiency, Chronic+" OR MH "Kidney+" OR TI(“dialysis” OR “Renal Dialyses” OR "Renal Dialysis" OR “Hemodialysis” OR “Hemodialyses” OR “Haemodialysis” OR “Haemodialyses” OR “hemofiltration” OR “haemofiltration” OR “Hemodiafiltration” OR “Haemodiafiltration” OR “kidney replacement therapy” OR “kidney replacement therapies” OR “renal replacement therapy” OR “renal replacement therapies” OR “kidney failure” OR “renal failure” OR “Chronic Kidney Diseases” OR “Chronic Kidney Disease” OR “Chronic Renal Diseases” OR “Chronic Renal Disease” OR “End Stage Kidney Disease” OR “End Stage Renal Disease” OR “renal insufficiency” OR “uraemia” OR “uremia” OR “azetoma” OR “ESKD” OR “ESRF” OR “ESKF” OR “CKD” OR “CRF” OR “CKF” OR “CRD” OR “KRT” OR “RRT”) OR AB(“dialysis” OR “Renal Dialyses” OR "Renal Dialysis" OR “Hemodialysis” OR “Hemodialyses” OR “Haemodialysis” OR “Haemodialyses” OR “hemofiltration” OR “haemofiltration” OR “Hemodiafiltration” OR “Haemodiafiltration” OR “kidney replacement therapy” OR “kidney replacement therapies” OR “renal replacement therapy” OR “renal replacement therapies” OR “kidney failure” OR “renal failure” OR “Chronic Kidney Diseases” OR “Chronic Kidney Disease” OR “Chronic Renal Diseases” OR “Chronic Renal Disease” OR “End Stage Kidney Disease” OR “End Stage Renal Disease” OR “renal insufficiency” OR “uraemia” OR “uremia” OR “azetoma” OR “ESKD” OR “ESRF” OR “ESKF” OR “CKD” OR “CRF” OR “CKF” OR “CRD” OR “KRT” OR “RRT”)**) AND (**MH "Vascular Access Devices+" OR MH "Arteriovenous Fistula" OR MH "Catheterization, Central Venous+" OR TI(“vascular access” OR “graft” OR “grafts” OR “fistula” OR “fistulas” OR “tunnelled catheter” OR “tunneled catheter” OR “nontunnelled catheter” OR “nontunneled catheter” OR “non tunnelled catheter” OR “non tunneled catheter” OR “central venous catheter” OR “central venous catheters” OR “vascular catheter” OR “vascular catheters” OR “venous catheter” OR “venous catheters” OR “central venous catheterization” OR “central venous catheterizations” OR “central venous catheterisation” OR “central venous catheterisations”) OR AB(“vascular access” OR “graft” OR “grafts” OR “fistula” OR “fistulas” OR “tunnelled catheter” OR “tunneled catheter” OR “nontunnelled catheter” OR “nontunneled catheter” OR “non tunnelled catheter” OR “non tunneled catheter” OR “central venous catheter” OR “central venous catheters” OR “vascular catheter” OR “vascular catheters” OR “venous catheter” OR “venous catheters” OR “central venous catheterization” OR “central venous catheterizations” OR “central venous catheterisation” OR “central venous catheterisations”)**)** **AND (**MH "Education+" OR MH "Health Education+" OR MH "Patient Education+" OR MH "Knowledge+" OR MH "Health Literacy" OR MH "Self Care+" OR MH "Self-Management" OR MH "Self-Efficacy" OR MH "Counseling+" OR TI(“educational” OR “education” OR “knowledge” OR “train*” OR “teach*” OR “treatment information” OR “health literacy” OR “behavioural” OR “behavioral” OR “behaviour” OR “behavior” OR “self management” OR “self care” OR “self efficacy” OR “cognitive” OR “counselling” OR “counseling”) OR AB(“educational” OR “education” OR “knowledge” OR “train*” OR “teach*” OR “treatment information” OR “health literacy” OR “behavioural” OR “behavioral” OR “behaviour” OR “behavior” OR “self management” OR “self care” OR “self efficacy” OR “cognitive” OR “counselling” OR “counseling”)**)** **AND (**LA English**)** **AND** PY 2013-2023 **NOT** ((MH "Animals+" OR MH "Animal Studies" OR TI animal model*) NOT MH "Human")

**APA PsycInfo (EBSCOhost)** 24 results
Includes **APA Thesaurus of Psychological Index Terms**

**(**DE "Hemodialysis" OR DE "Kidneys" OR TI(“dialysis” OR “Renal Dialyses” OR "Renal Dialysis" OR “Hemodialysis” OR “Hemodialyses” OR “Haemodialysis” OR “Haemodialyses” OR “hemofiltration” OR “haemofiltration” OR “Hemodiafiltration” OR “Haemodiafiltration” OR “kidney replacement therapy” OR “kidney replacement therapies” OR “renal replacement therapy” OR “renal replacement therapies” OR “kidney failure” OR “renal failure” OR “Chronic Kidney Diseases” OR “Chronic Kidney Disease” OR “Chronic Renal Diseases” OR “Chronic Renal Disease” OR “End Stage Kidney Disease” OR “End Stage Renal Disease” OR “renal insufficiency” OR “uraemia” OR “uremia” OR “azetoma” OR “ESKD” OR “ESRF” OR “ESKF” OR “CKD” OR “CRF” OR “CKF” OR “CRD” OR “KRT” OR “RRT”) OR AB(“dialysis” OR “Renal Dialyses” OR "Renal Dialysis" OR “Hemodialysis” OR “Hemodialyses” OR “Haemodialysis” OR “Haemodialyses” OR “hemofiltration” OR “haemofiltration” OR “Hemodiafiltration” OR “Haemodiafiltration” OR “kidney replacement therapy” OR “kidney replacement therapies” OR “renal replacement therapy” OR “renal replacement therapies” OR “kidney failure” OR “renal failure” OR “Chronic Kidney Diseases” OR “Chronic Kidney Disease” OR “Chronic Renal Diseases” OR “Chronic Renal Disease” OR “End Stage Kidney Disease” OR “End Stage Renal Disease” OR “renal insufficiency” OR “uraemia” OR “uremia” OR “azetoma” OR “ESKD” OR “ESRF” OR “ESKF” OR “CKD” OR “CRF” OR “CKF” OR “CRD” OR “KRT” OR “RRT”)**) AND (**TI(“vascular access” OR “graft” OR “grafts” OR “fistula” OR “fistulas” OR “tunnelled catheter” OR “tunneled catheter” OR “nontunnelled catheter” OR “nontunneled catheter” OR “non tunnelled catheter” OR “non tunneled catheter” OR “central venous catheter” OR “central venous catheters” OR “vascular catheter” OR “vascular catheters” OR “venous catheter” OR “venous catheters” OR “central venous catheterization” OR “central venous catheterizations” OR “central venous catheterisation” OR “central venous catheterisations”) OR AB(“vascular access” OR “graft” OR “grafts” OR “fistula” OR “fistulas” OR “tunnelled catheter” OR “tunneled catheter” OR “nontunnelled catheter” OR “nontunneled catheter” OR “non tunnelled catheter” OR “non tunneled catheter” OR “central venous catheter” OR “central venous catheters” OR “vascular catheter” OR “vascular catheters” OR “venous catheter” OR “venous catheters” OR “central venous catheterization” OR “central venous catheterizations” OR “central venous catheterisation” OR “central venous catheterisations”)**)** **AND (**DE "Education" OR DE "Client Education" OR DE "Consumer Education" OR DE "Health Education" OR DE "Knowledge (General)" OR DE "Health Knowledge" OR DE "Health Literacy" OR DE "Self-Care" OR DE "Self-Management" OR DE "Self-Efficacy" OR DE "Counseling" OR TI(“educational” OR “education” OR “knowledge” OR “train*” OR “teach*” OR “treatment information” OR “health literacy” OR “behavioural” OR “behavioral” OR “behaviour” OR “behavior” OR “self management” OR “self care” OR “self efficacy” OR “cognitive” OR “counselling” OR “counseling”) OR AB(“educational” OR “education” OR “knowledge” OR “train*” OR “teach*” OR “treatment information” OR “health literacy” OR “behavioural” OR “behavioral” OR “behaviour” OR “behavior” OR “self management” OR “self care” OR “self efficacy” OR “cognitive” OR “counselling” OR “counseling”)**)** **AND** **(**LA English**)** **AND** (PY 2013-2023)

*Link to saved search for Cochrane -* [*https://www.cochranelibrary.com/advanced-search/search-manager?search=7216115*](https://www.cochranelibrary.com/advanced-search/search-manager?search=7216115)

**Cochrane Library (Wiley)** 15 results

Includes MeSH

Advanced search > Search manager


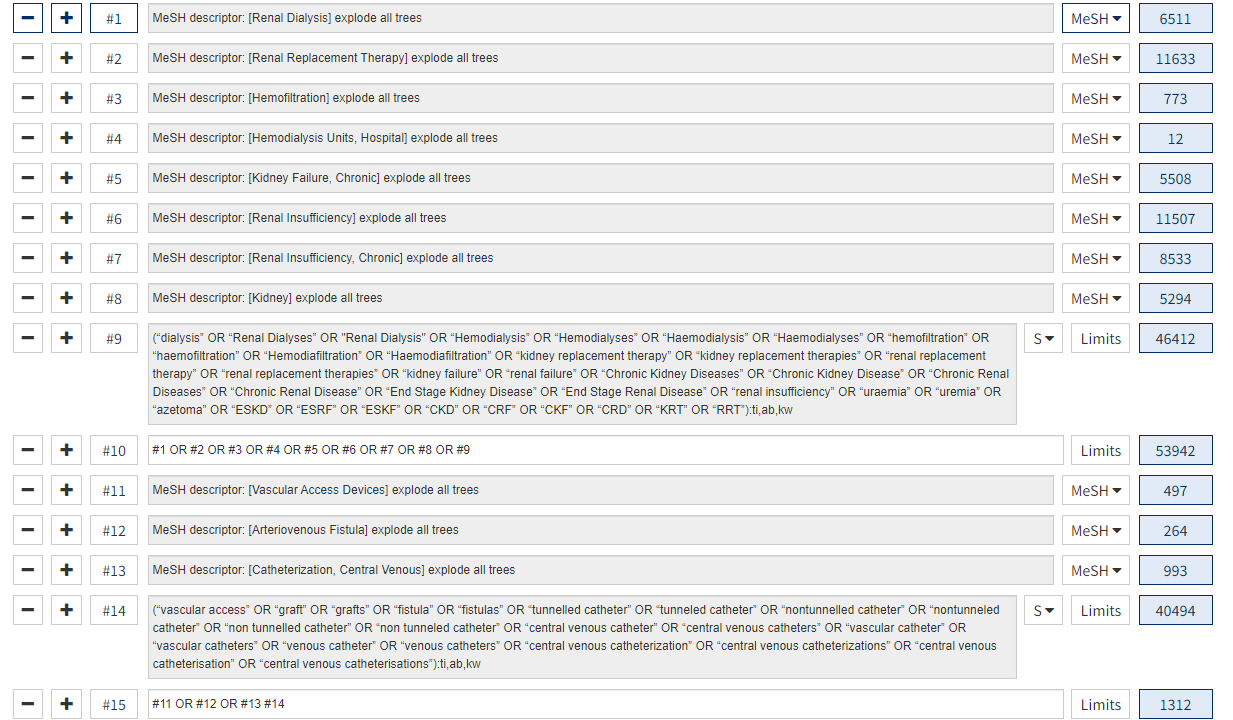


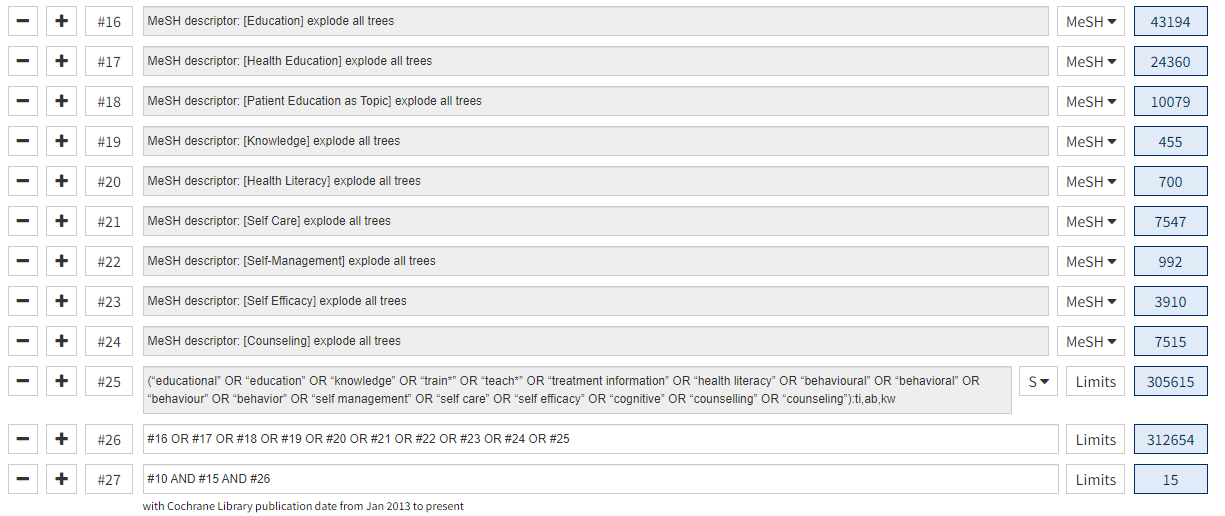


ID Search Hits

#1 MeSH descriptor: [Renal Dialysis] explode all trees 6511

#2 MeSH descriptor: [Renal Replacement Therapy] explode all trees 11633

#3 MeSH descriptor: [Hemofiltration] explode all trees 773

#4 MeSH descriptor: [Hemodialysis Units, Hospital] explode all trees 12

#5 MeSH descriptor: [Kidney Failure, Chronic] explode all trees 5508

#6 MeSH descriptor: [Renal Insufficiency] explode all trees 11507

#7 MeSH descriptor: [Renal Insufficiency, Chronic] explode all trees 8533

#8 MeSH descriptor: [Kidney] explode all trees 5294

#9 (“dialysis” OR “Renal Dialyses” OR "Renal Dialysis" OR “Hemodialysis” OR “Hemodialyses” OR “Haemodialysis” OR “Haemodialyses” OR “hemofiltration” OR “haemofiltration” OR “Hemodiafiltration” OR “Haemodiafiltration” OR “kidney replacement therapy” OR “kidney replacement therapies” OR “renal replacement therapy” OR “renal replacement therapies” OR “kidney failure” OR “renal failure” OR “Chronic Kidney Diseases” OR “Chronic Kidney Disease” OR “Chronic Renal Diseases” OR “Chronic Renal Disease” OR “End Stage Kidney Disease” OR “End Stage Renal Disease” OR “renal insufficiency” OR “uraemia” OR “uremia” OR “azetoma” OR “ESKD” OR “ESRF” OR “ESKF” OR “CKD” OR “CRF” OR “CKF” OR “CRD” OR “KRT” OR “RRT”):ti,ab,kw 46412

#10 #1 OR #2 OR #3 OR #4 OR #5 OR #6 OR #7 OR #8 OR #9 53942

#11 MeSH descriptor: [Vascular Access Devices] explode all trees 497

#12 MeSH descriptor: [Arteriovenous Fistula] explode all trees 264

#13 MeSH descriptor: [Catheterization, Central Venous] explode all trees 993

#14 (“vascular access” OR “graft” OR “grafts” OR “fistula” OR “fistulas” OR “tunnelled catheter” OR “tunneled catheter” OR “nontunnelled catheter” OR “nontunneled catheter” OR “non tunnelled catheter” OR “non tunneled catheter” OR “central venous catheter” OR “central venous catheters” OR “vascular catheter” OR “vascular catheters” OR “venous catheter” OR “venous catheters” OR “central venous catheterization” OR “central venous catheterizations” OR “central venous catheterisation” OR “central venous catheterisations”):ti,ab,kw 40494

#15 #11 OR #12 OR #13 #14 1312

#16 MeSH descriptor: [Education] explode all trees 43194

#17 MeSH descriptor: [Health Education] explode all trees 24360

#18 MeSH descriptor: [Patient Education as Topic] explode all trees 10079

#19 MeSH descriptor: [Knowledge] explode all trees 455

#20 MeSH descriptor: [Health Literacy] explode all trees 700

#21 MeSH descriptor: [Self Care] explode all trees 7547

#22 MeSH descriptor: [Self-Management] explode all trees 992

#23 MeSH descriptor: [Self Efficacy] explode all trees 3910

#24 MeSH descriptor: [Counseling] explode all trees 7515

#25 (“educational” OR “education” OR “knowledge” OR “train*” OR “teach*” OR “treatment information” OR “health literacy” OR “behavioural” OR “behavioral” OR “behaviour” OR “behavior” OR “self management” OR “self care” OR “self efficacy” OR “cognitive” OR “counselling” OR “counseling”):ti,ab,kw 305615

#26 #16 OR #17 OR #18 OR #19 OR #20 OR #21 OR #22 OR #23 OR #24 OR #25 312654

#27 #10 AND #15 AND #26 with Cochrane Library publication date from Jan 2013 to present 15

**JBI EBP database (Ovid)** 2 results

Advanced search

("dialysis".ti,ab. OR "Renal Dialyses".ti,ab. OR "Renal Dialysis".ti,ab. OR "Hemodialysis".ti,ab. OR "Hemodialyses".ti,ab. OR "Haemodialysis".ti,ab. OR "Haemodialyses".ti,ab. OR "hemofiltration".ti,ab. OR "haemofiltration".ti,ab. OR "Hemodiafiltration".ti,ab. OR "Haemodiafiltration".ti,ab. OR "kidney replacement therapy".ti,ab. OR "kidney replacement therapies".ti,ab. OR "renal replacement therapy".ti,ab. OR "renal replacement therapies".ti,ab. OR "kidney failure".ti,ab. OR "renal failure".ti,ab. OR "Chronic Kidney Diseases".ti,ab. OR "Chronic Kidney Disease".ti,ab. OR "Chronic Renal Diseases".ti,ab. OR "Chronic Renal Disease".ti,ab. OR "End Stage Kidney Disease".ti,ab. OR "End Stage Renal Disease".ti,ab. OR "renal insufficiency".ti,ab. OR "uraemia".ti,ab. OR "uremia".ti,ab. OR "azetoma".ti,ab. OR "ESKD".ti,ab. OR "ESRF".ti,ab. OR "ESKF".ti,ab. OR "CKD".ti,ab. OR "CRF".ti,ab. OR "CKF".ti,ab. OR "CRD".ti,ab. OR "KRT".ti,ab. OR "RRT".ti,ab.) AND ("vascular access".ti,ab. OR "graft".ti,ab. OR "grafts".ti,ab. OR "fistula".ti,ab. OR "fistulas".ti,ab. OR "tunnelled catheter".ti,ab. OR "tunneled catheter".ti,ab. OR "nontunnelled catheter".ti,ab. OR "nontunneled catheter".ti,ab. OR "non tunnelled catheter".ti,ab. OR "non tunneled catheter".ti,ab. OR "central venous catheter".ti,ab. OR "central venous catheters".ti,ab. OR "vascular catheter".ti,ab. OR "vascular catheters".ti,ab. OR "venous catheter".ti,ab. OR "venous catheters".ti,ab. OR "central venous catheterization".ti,ab. OR "central venous catheterizations".ti,ab. OR "central venous catheterisation".ti,ab. OR "central venous catheterisations".ti,ab.) AND ("educational".ti,ab. OR "education".ti,ab. OR "knowledge".ti,ab. OR "train*".ti,ab. OR "teach*".ti,ab. OR "treatment information".ti,ab. OR "health literacy".ti,ab. OR "behavioural".ti,ab. OR "behavioral".ti,ab. OR "behaviour".ti,ab. OR "behavior".ti,ab. OR "self management".ti,ab. OR "self care".ti,ab. OR "self efficacy".ti,ab. OR "cognitive".ti,ab. OR "counselling".ti,ab. OR "counseling".ti,ab.)


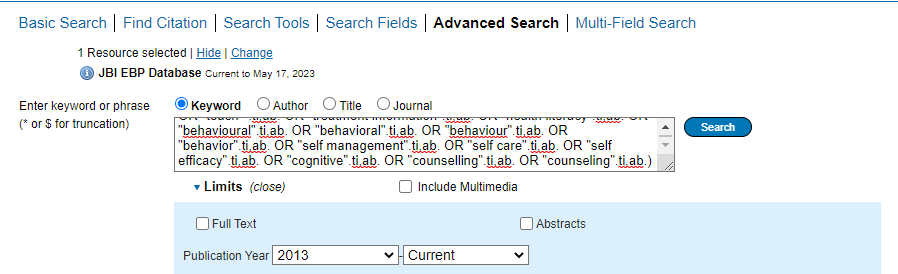


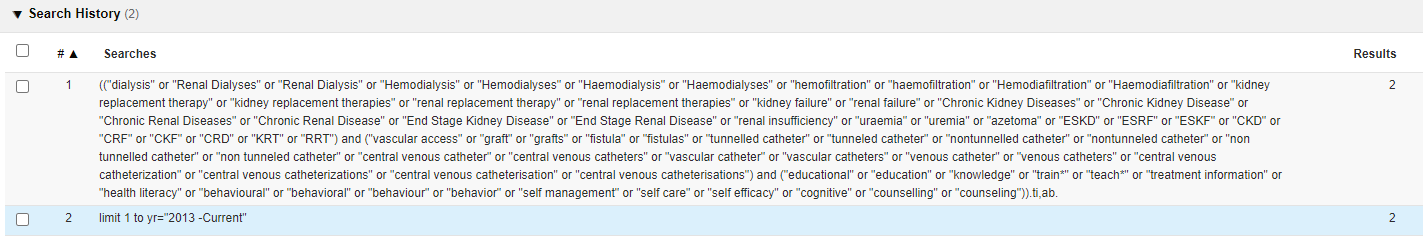


*Link to search -* [*https://ovidsp.ovid.com/ovidweb.cgi?T=JS&NEWS=N&PAGE=main&SHAREDSEARCHID=bgtcgXeA3QZ6ZTc16jj4sHxOgSw1htmq3atNZjaReJFXFG6Ciblh5WcpKSizvkME*](https://ovidsp.ovid.com/ovidweb.cgi?T=JS&NEWS=N&PAGE=main&SHAREDSEARCHID=bgtcgXeA3QZ6ZTc16jj4sHxOgSw1htmq3atNZjaReJFXFG6Ciblh5WcpKSizvkME)
