# Supplementary material for: Educational or Behavioural Interventions to Improve Long‐Term Haemodialysis Vascular Access Self‐Management: A Systematic Review
Source: J Ren Care. 2025 Jan 28;51(1):e70005. doi: 10.1111/jorc.70005 (PMC11774008; doi:10.1111/jorc.70005)
Supplement: Supplementary file 2 — Supporting information. [file JORC-51-0-s001.docx]

**Supplementary 2: Critical appraisal of included studies**

**Quasi-experimental studies**

| Questions | Borzou et al. (2020) | Fadlalmola & Ekareem (2020) | Li & Yin (2021) | Sousa et al. (2021) | Trask et al. (2016) |
| --- | --- | --- | --- | --- | --- |
| Q1. Is it clear in the study what is the ‘cause’ and what is the ‘effect’ (i.e., there is no confusion about which variable comes first)? | U | Y | Y | Y | U |
| Q2. Were the participants included in any comparisons similar? | Y | Y | U | Y | Y |
| Q3. Were the participants included in any comparisons receiving similar treatment/care, other than the exposure or intervention of interest? | Y | Y | Y | Y | Y |
| Q4. Was there a control group? | N | N | Y | Y | N |
| Q5. Were there multiple measurements of the outcome both pre and post the intervention/exposure? | Y | Y | Y | Y | Y |
| Q6. Was follow-up complete and if not, were differences between groups in terms of their follow-up adequately described and analysed? | Y | U | U | Y | Y |
| Q7. Were the outcomes of participants included in any comparisons measured in the same way? | Y | Y | Y | Y | Y |
| Q8. Were outcomes measured in a reliable way? | Y | Y | U | Y | Y |
| Q9. Was appropriate statistical analysis used? | Y | N | N | Y | Y |
| % of criteria met | 78% | 67% | 56% | 100% | 78% |

Note: Y=Yes; N=No; U=Unclear; NA=Not Applicable due to study design

Source: JBI Critical Appraisal Checklist for Quasi-Experimental Studies (non-randomized experimental studies)

Available from <https://jbi.global/critical-appraisal-tools>

**Randomized Controlled Trials**

| Questions | Liu et al. (2016) | Ramezani et al. (2019) |
| --- | --- | --- |
| Q1. Was true randomization used for assignment of participants to treatment groups? | Y | U |
| Q2. Was allocation to treatment groups concealed? | U | U |
| Q3. Were treatment groups similar at the baseline? | Y | Y |
| Q4. Were participants blind to treatment assignment? | NA | NA |
| Q5. Were those delivering treatment blind to treatment assignment? | NA | NA |
| Q6. Were outcomes assessors blind to treatment assignment? | U | U |
| Q7. Were treatment groups treated identically other than the intervention of interest? | Y | Y |
| Q8. Was follow-up complete and if not, were differences between groups in terms of their follow-up adequately described and analyzed? | Y | Y |
| Q9. Were participants analyzed in the groups to which they were randomized? | U | N |
| Q10. Were outcomes measured in the same way for treatment groups? | Y | Y |
| Q11. Were outcomes measured in a reliable way? | Y | Y |
| Q12. Was appropriate statistical analysis used? | N | N |
| Q13. Was the trial design appropriate, and any deviations from the standard RCT design (individual randomization, parallel groups) accounted for in the conduct and analysis of the trial? | Y | Y |
| % of criteria met | 64% | 55% |

Note: Y=Yes; N=No; U=Unclear; NA=Not Applicable due to study design

Source: JBI Critical Appraisal Checklist for Randomized Controlled Trials

Available from <https://jbi.global/critical-appraisal-tools>
